# The Conquest*President’s Address, Kansas State Homœopathic Society, 1896.

**Published:** 1896-07

**Authors:** C. F. Menninger

**Affiliations:** Topeka, Kansas


### THE CONQUEST.*

C. F. MENNINGER, M. D., TOPEKA, KANSAS.

Truth and error have been in contest ever since the world began. Throughout the history of the human race we behold the struggle for supremacy between right and wrong. Here we see the forces of darkness and destruction gain seeming victory; there the hosts of light are triumphant. This ceaseless conquest is still going on. Ignorance, error, darkness, vice, degradation, and crime—all these in fact the same, in seeming only, different—are waging war against truth, intelligence, light, virtue, and happiness.

To confine our scope of view to the limits of but a few years would render our judgment imperfect. To adequately judge of the true state of affairs in this conquest centuries of time are none too long. He who mounts the heights and overlooks the whole valley of time that stretches out before him into the dim unknown, and can stand there with a clear brain, an unsullied conscience, and a pure heart, can see through all the maze of human action, as recorded in history, sacred and profane, the progress of right against wrong, the conquest of truth over error. He can see the self-destruction of evil, the suicide of darkness, and the vanquished form of crime, all because of the victories of truth, of right, of intelligence.

The world is growing better, is growing more intelligent, is growing happier. The steady advance of all peoples from ignorance to intelligence, from misery to happiness, from barbarity to civilization, demonstrates irrefutably the final and absolute victory of truth.

*Divine Love* is the motive, *Infinite Wisdom* the power, and *Education* the means whereby mankind will be able to attain to that perfection. "*For God so loved the world,*" is a message so

---

* President's Address, Kansas State Homœopathic Society, 1896.

full of tender feeling as to touch the hearts of all who hear it. It is the motive that gives us life and happiness; that surrounds us with all that charms the ear and delights the eye in wondrous nature, in all her myriad forms and moods.

Infinite Wisdom is the power that makes and unmakes the laws of the universe. It started and keeps moving the wondrous world of stars and planets. It rides upon the winds and flashes with the lightning. Wisdom in everything, as laws that know no changing speak in clarion tones the infiniteness of God. On all hands He reveals Himself to us, if *we* will but see. If *we* but *inform* ourselves of His works and through them of Himself, we will attain more and more *like unto Him.*

*Education* is the means whereby we may be able to enjoy these blessings. It is the key that unlocks the gates which shut out happiness from mankind. It is the light and life of the nation. Through education, truth and right will conquer. The conquest of civilization over barbarism, of Christianity over heathenism, of science over chance, will alone be won by education.

It was through education that Homœopathy was brought into the world. Although known in the dim, dark ages, the law of similars was announced to the world as the only law of cure by one of the most successful and best educated physicians of the last century. Homœopathy could never have come into existence as it is to-day except by the guidance of so able a man as Samuel Hahnemann, and through the efforts of his scholarly pupils. These men had obtained a scientific and classical education in the best Universities of the continent; they had completed, as post-graduates, the study of the science of medicine as it was then taught by the highest and best authorities. But they found the allopathic system of medicine wholly inadequate, or, rather, they found the system of medicine founded by Hahnemann far superior in every respect. They deserted Allopathy and embraced Homœopathy with such fervor and devotion as is rarely found. The mantle of shame and disgrace was laid upon them, and with abusive and defamatory words they were driven almost into exile. Yet they had found the truth and to this they adhered.

Education is the handmaid of Homœopathy, for by it this system of medicine was ushered into the world; by it Homœopathy has made its stupendous growth in face of the bitterest opposition, and it is by education alone that Homœopathy will be able to conquer the world.

One of the most serious drawbacks that Homœopathy has had, and this especially so in the last half of this century, has been the uneducated practitioner. There have been and are in the ranks of Homœopathy men and women as practitioners, as exponents of the immortal Hahnemann and his magnificent system of medicine, who could not pass a creditable examination in the common-school branches. There are many who, on account of the lack of education, made a glorious failure as teachers, ministers, and lawyers who have entered the ranks of Homœopathy as practitioners. This is not the class of followers Hahnemann had as his first disciples. Incompetent men and women have brought the homœopathic school of medicine into disrepute. Having little knowledge of the rudiments of medicine, and still less of the philosophy of Homœopathy, they cause strife as to the correct application of this law of cure. The best educated physicians of the homœopathic school, not only in a scientific way, but also in all that appertains to medicine, its collateral branches, and especially in the philosophy of Homœopathy, its history and literature, are strict followers of Hahnemann and his earliest disciples. They are Hahnemannian homœopaths. I challenge any refutation of this assertion.

The homœopathic profession has recognized that education is the handmaid of Homœopathy. Practical experience and observation have taught that the best patients in every sense of the word, and the best supporters in spreading the gospel of truth, are the educated patrons. The National Society has expressed itself in favor of education by increasing the length of term in college, and by demanding of the matriculant greater proficiency before he can begin his medical studies.

Homœopathy has done more for the people than any other system of medicine by educating the masses. It encourages investigation; invites parents to procure family medicine cases and books from which to study its philosophy and art of application. Homœopathy writes its prescriptions in common, everyday vernacular and discourages mysticism in every way. Homœopathy is synonymous *with light*, not darkness; *with truth*, not error; *with intelligence*, not ignorance. It encourages and invites investigation. Homœopathy wants all to "*reach hither thy finger*," to investigate by actual experiment. It does not suffer by having men doubt its efficacy. It matters not how unreasonable they may be, if they are only honest and will lay aside prejudice long enough to investigate. There is a large class of people who are so narrow and prejudiced and so opposed to all investigation, that it is almost impossible to get them to entertain one favorable thought toward our system of medicine. They will not allow themselves to approach the question.

It is a hard matter to change a man's ideas and methods after they have once become established in his mind. Prejudice is often unbounded. It will not allow him to see the good results of Homœopathy. He will not do as Dr. Hering did, "READ IT UP TO WRITE IT DOWN." Dr. Hering was an honest man. He believed Homœopathy was wrong, and in an honest way he went about to prove it to be false. He began to study it thoroughly so that he might PROVE IT *to be false*, but in that investigation he became CONVINCED *of the truth of the homœopathic law.* So much for honest investigation.

Homœopathy is capable of being clearly demonstrated to any thoughtful mind. It rests upon *facts* which can be and are being multiplied any number of times, and which cannot by any process of reasoning be set aside. We may not be able to satisfactorily explain how the small dose cures, but the *fact* nevertheless remains *that it does cure.* "Nothing can be known with certainty except by an appeal to facts." This is inductive reasoning, by means of which Hahnemann was able to arrive at the law of cure underlying all observable phenomena in therapeutics. This is the first great step in the process without which man can never be certain that he knows anything. Therefore

Hering, fully appreciating this, said : "If our school ever gives up the strict inductive method of Hahnemann, we are lost, and deserve only to be mentioned as a caricature in the history of medicine."

Yet there are those, no small number, who declare Homœopathy to be a myth ; that the benefits experienced by hundreds of thousands of its patrons are absurdities ; who decry its merits and virtues ; who deny it a place in the world's history of scientific attainments. This is but one example to which we have alluded, namely, that there is a ceasless strife going on between ignorance and intelligence, truth and error, light and darkness. Each is struggling for supremacy. The conquest is between Homœopathy and all those who oppose it. Homœopathy is intelligence, is light, is truth. The opposition is ignorance, is darkness, is error. Homœopathy invites you to investigate. The opposition refuses flatly, and without foundation in fact declares it unreasonable. Homœopathy offers itself in love. The opposition rejects it with bigotry and hate. The conquest is on. It is a fight to the finish. As in battle the private, and not the officer, does the effective work that brings decision and wins the victory, so it is the general practitioner in his humble position who *alone* is capable to lead *us on to victory.* No small responsibility rests upon his shoulders. Fully equipped with all that education can give him he further needs *energy, courage, perseverance,* and a heart illumed with DIVINE LOVE and TRUTH. In his own field of practice the battle must be fought. It seems to me that the three most important points that will require his greatest attention, are 1st, *to practice pure Homœopathy ;* 2d, *to educate the people in Homœopathy ;* and 3d, *to encourage only those who are competent, and discourage all incompetent from studying medicine.*

To practice pure Homœopathy he must be a close student and follower of Hahnemann, and such disciples as Jahr, Bœnninghausen, Lippe, Dunham, and Hering. The *practice* of these men built the enduring system of medicine which we have chosen. Under the most trying circumstances of social and professional ostracism and persecution, with steadfastness of purpose to do right, they, as privates in the rank of battle, carried on the fight. Through the greatest of adversity they established our colors, and held the fort against all assaults. Their thoughts and their works are surely fit objects of study, and worthy of being imitated.

But have we not learned better ways since then? Would they not have modified their rules and kept abreast with modern methods had they lived to-day? No. *A law of nature once established is as firmly fixed and as unchangeable as a proposition in mathematics; is as true to-day as it was at its foundation.* THERE CAN BE NO REVERSAL.

The law of similars was established by inductive reasoning. By the accurate observation of well-authenticated facts Hahnemann and his able disciples established the science of therapeutics. All that has been achieved in the progress of medical science since that day has been but additional data to confirm this law. The laws governing the motions of the stars and planets have been confirmed since the days of Newton and Kepler. Not one single change has been made necessary by the addition of new evidence. Yet the science of astronomy has improved since those days. So with the science of therapeutics. All new evidence, all new discoveries, have not made it necessary to make a single change in the law of similars.

All the phenomena of drug action and of signs of disease that have been discovered and demonstrated subsequent to the formulation of the law of similars *harmonize perfectly with the terms of this law;* and yet the science of medicine has improved since those days.

Those who say that it is necessary to so change Homœopathy as to keep up with the times, or that they are modern homœopaths, are unmindful of the fact that the law of similars is as much a law of nature to-day as it was in the days of Hahnemann, and, *as such*, as unchangeable as any other law of nature. Who ever heard of modern physicists who have improved upon the law of gravitation since the days of Newton? Who thinks that the law must be modernized in order to suit the times? That principle which is capable of indefinite expansion and elaboration without in the least altering it, is alone worthy of being classed as a law of nature; and such a principle alone will answer the purposes upon which to construct a science.

Had the practice of the men cited been founded on speculative grounds, as is that of the allopaths to-day, it would have been subject to reaction. Speculative science has no permanence. Inductive science is eternal. "A fact is a fact yesterday, to-day, and forever. If like cures like because it is the dictum of unchanging nature that it should, then there can be no change, however much the sluggard would have it so."

Every homœopathic physician has demonstrated to his fullest satisfaction that the potentized drug cures diseases, not once, but all the time, under similar circumstances. Shall we turn from that certainty and trifle with uncertainties? Every one who has tried it knows that the single remedy bears more intelligent evidence than the multiple. Shall we grope 'mid the uncertainty of alternation? We know that disease attacks in varying force, and that varying force is required to combat it. Then shall we wield the sledge in every case when the gentler vibration of insensible molecular motion would be sufficient? In other words, is it not best to use the lowest to highest potencies compatible with cures?

We must use *the pure homœopathic practice in order to win the victory.* This calls for *the similar remedy, the single remedy,* and *the minimum dose.* "With this mode of cure there can be no compromise. Homœopathy is either dead right or dead wrong, it is everything or it is nothing."

Besides practicing pure Homœopathy, which I regard as the first and most important step to take, to conquer the world for Homœopathy, there remains other things for the general practitioner to do. We would be false to our profession, indifferent to the welfare of the sick, and regardless of the fame of him to whom, in therapeutics, we owe nearly everything, were we to neglect to enlighten the public. Homœopathy is in many places not even known; in others most imperfectly, and therefore the butt of ridicule and derision. Among the members of the medical profession not of our school there exists a woeful lack of knowledge. Much to their discredit there exists also a determination not to inform themselves or institute investigation, and they give utterance to opinions detrimental to Hahnemann and contemptuous of Homœopathy without possessing even a modicum amount of knowledge of the same. The public does not know that they are so ignorant, and hence can scarcely believe it when told, for they look upon the profession as the fountain of all medical knowledge. It is our duty to set forth the true status of Hahnemann, of his great and transcendent merits as an original observer, as a patient and careful therapeutic investigator, and as a scientific thinker. We must show by comparison the vast superiority of the homœopathic practice over the allopathic. By informing the public of what Homœopathy means and of the results which accrue from it, we obtain powerful assistance in making yet more generally serviceable the important therapeutic truths of which we are the trustees.

In his own private practice the general practitioner has yet another duty to perform in order that he may render his best service toward the conquest of Homœopathy. It is one which requires much study and deliberation and which will ofttime tax all his powers to perform faithfully and adequately. But if he has at heart the true love of his profession and the highest regard for humanity he will not fail to encourage only those who are competent and discourage all incompetent from studying medicine. It requires an unlimited amount of unusually good common sense and discretion to do this, yet it is a duty which, when neglected, causes untold damage; when performed with tact, it brings forth invaluable aid in the conquest.

Thus far the work of the general practitioner has been confined to his own field of practice. So far he has worked by himself. But he has associates in different parts of the country, and with them jointly he can and must do much to secure final victory. Frequently this part of the work is neglected by the many, and then it falls to the few. *In order that we may conquer this State for Homœopathy, we must unite with our fellow-*

*workers in organized movements for the advancement of our cause. Our State society needs new life.* It has muscle and bone and sinew enough to be a power. But there is an apathy, an indifference, a don't-care-sort-of-a-feeling that has taken possession of its members which needs to be shaken off. We must arouse ourselves from this dreamy state and go forth to win.

We need more and better organization. In addition to having one State society there ought to be organized at least three sectional societies, a central, a southeastern, and a northeastern Homœopathic Medical Society. These meetings could be so timed as to not interfere with the workings of one another or with the parent society of the State. If we would amend our Constitution so as to elect three Vice-Presidents, one from each of these three sections of the State, and let these Vice-Presidents be the Presidents of the sectional societies, much good for the cause could be done by way of thorough co-operation. In order to do the most effective educational and missionary work for Homœopathy, it might be well to observe and consider the organization and operation of successful associations, such as the religious and educational societies of this and other States. See how they are organized and conducted. Surely our work and our cause is no less important nor less inspiring than theirs. They are fit bodies for our emulation. We cannot and must not rest upon our arms in this apathetic way. The progress or regress of Homœopathy depends upon its representatives. WE MUST GO TO WORK.

Through organized efforts only will we be able to demand and obtain recognition from authorities who have denied it thus far. This body of homœopathic physicians here assembled owes it as a duty to the whole world of homœopathic physicians and their patrons, and to the large number of people in this State who prefer this practice, that we leave *nothing undone for the good of this cause.* It is demanded of us that we put forth every effort to advance Homœopathy in this State; that we obtain charge of the medical department of all the State charitable institutions; that we obtain a voice in all public or semipublic questions which affect their health or well-being; that we so associate ourselves as to most effectively and in the most lasting way obtain the confidence, good-will, and approbation of all the people of this State. This is demanded of us because we have voluntarily taken upon ourselves the care and keeping of the sacred truths of Homœopathy. We are responsible for making known these truths, and for their universal acceptance, because we, of our own free will, have chosen to be their trustees.

Nor do the boundary lines of this State limit the sphere of our duties. We have no right to be independent of all others. We must exert our influence within our domain so as to have its influence and power extend all over the civilized world. That we may conquer in this fight, we must express our approval or disapproval of all things that relate to the progress of our school. It is our duty as members of the great body of the homœopathic fraternity to see that everything is being done to advance it throughout the world. It therefore becomes necessary for the general practitioner to decide what instruction medical colleges should give and how patients should be treated in our hospitals. We must demand of all the colleges the best instruction for students in the science and art of medicine and surgery, and especially in Homœopathy. This instruction must be given by the best teachers obtainable. It is not alone necessary that he be a success as a practitioner, he must have talent as an instructor and such attributes as go to make a man. Now as to the subject-matter taught: As homœopathic practitioners *we must demand the teaching of* HOMŒOPATHY. This is the crying need of the day in our medical schools. There are but very few schools in this country where anything at all adequate in this respect is the subject of instruction. Most of our so-called homœopathic medical colleges are guilty of a crime for taking the money of students and promising to give them certain instruction and then absolutely failing to do so. They give a medical education, as good as can be obtained in any college in the world, but the *student of Homœopathy* went there to get a HOMŒOPATHIC medical education. The addition of that word means certainly something, and is not used merely as a catch-penny phrase. If it does not mean anything, then the sooner it is left out of announcements the higher will be the respect entertained for the college. But, if it is to be left in the announcements, and they really mean *to give* and *to do* what they say, nine-tenths of our medical colleges need *to begin to teach Homœopathy.* Homœopathy is not everything in medicine, *but it is everything to the* HOMŒOPATHIC PRACTITIONER. Our students need to be *filled with Homœopathy, its history, its laws, its literature, its great exponents and their ideas, its mode of application—in short, with all that pertains to this science that the immortal Hahnemann gave to the world. It must be taught from the opening until the final* GRADUATION DAY. *It must be taught by the best instructors obtainable. This is the distinctive feature of our school.* To US *it means* EVERYTHING. *Therefore the absolute necessity of its thorough instruction.*

A homœopathic hospital is an establishment for the cure and alleviation of the sick and suffering upon the principles enunciated and promulgated by the founder of the homœopathic art. Anything short of it, or contrary to it, is a species of fraud and a betrayal of a trust, both private and public. If they are conducted upon the principles of Homœopathy they will exhibit three sets of facts, all of which show the superiority of our school of medicine over every other, any one of which would be inducement and reason sufficient to decide the question of the value of our system of medicine. These three sets of facts are: 1, Homœopathic treatment shortens the time of sickness in contrast with any other mode; 2, Homœopathy reduces the mortality below that of all other systems or modes of cure; and 3, Homœopathy reduces the expenses of both the patient and institution far below that of all other systems of medicine. These three points commend themselves to the intelligence and sound judgment of all thinking people. That we may continue to do this in the fullest sense *the general practitioner of our State must see to it that all our hospitals be conducted upon homœopathic principles, and* NONE OTHER. The responsibility lies with the entire profession. If it will suffer them to be conducted upon any other basis it is a participant in the fraud perpetrated.

If we would extend the domain of Homœopathy throughout the world, if we would conquer this State for Homœopathy, the campaign as mapped out must, in a large measure, be the plan of action. The work must be done by the general practitioner in his private practice by using pure Homœopathy, by educating the people, by encouraging only those who are competent to study medicine; throughout the State, in associating himself with his fellow-practitioners in more thorough organization, and through them demanding public recognition; throughout the world he must exercise a censorship over our colleges and our hospitals, *commending what is right and condemning everything detrimental to our art of cure. These are self-imposed duties to which he cannot prove recreant unless he violate a sacred trust.*

